# Supplementary material for: Neuralized-like proteins differentially activate Notch ligands
Source: EMBO Rep. 2025 Oct 31;26(23):5756–75. doi: 10.1038/s44319-025-00601-7 (PMC12678834; doi:10.1038/s44319-025-00601-7)
Supplement: Supplementary file 1 — Appendix [file 44319_2025_601_MOESM1_ESM.pdf]

Appendix Figures

Table of Contents

Appendix Figure S1 2

Appendix Figure S2 3

Appendix Figure S3 4

Appendix Figure S4 5

Appendix Figure S5 6

Appendix Figure S1

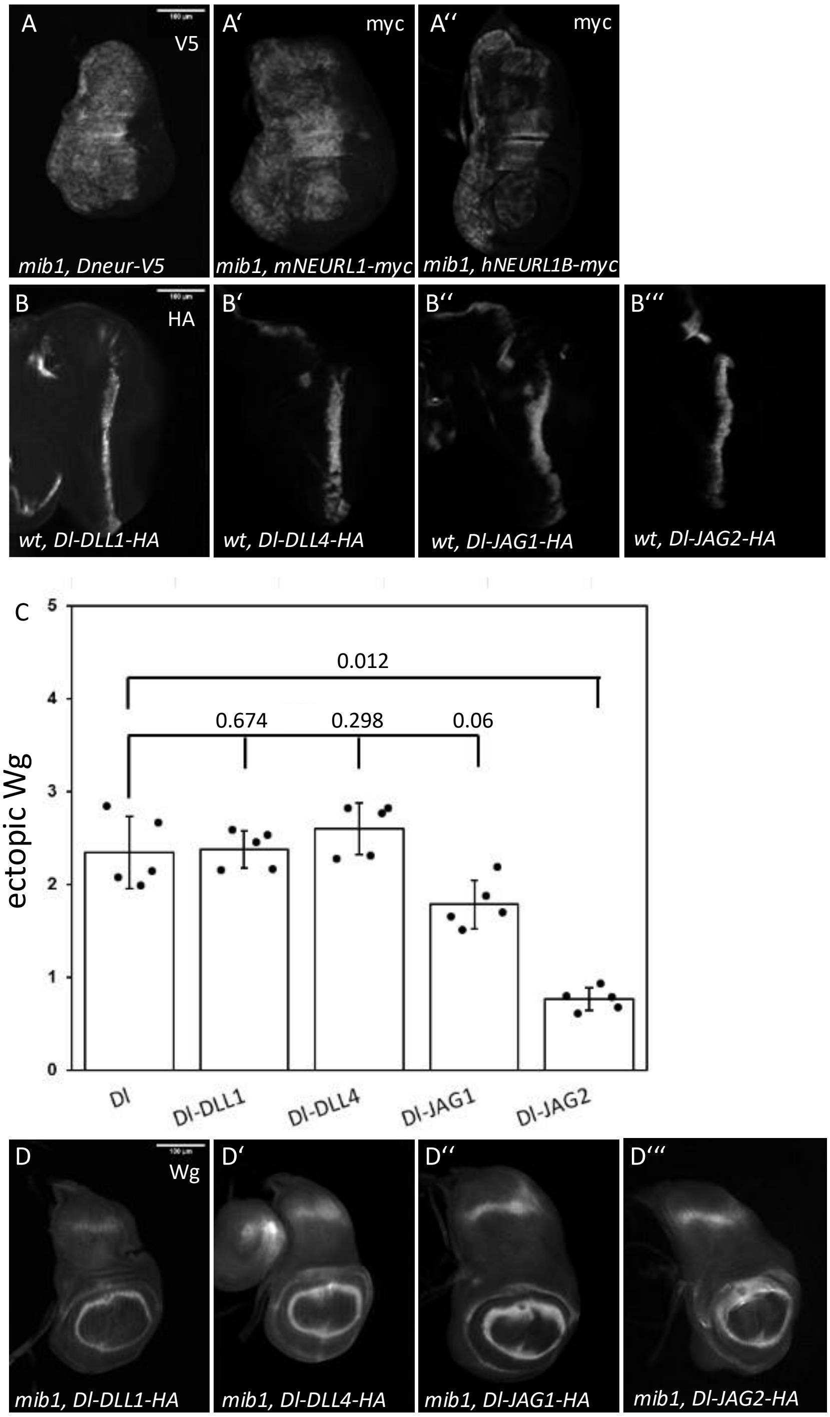

Appendix Fig. S1. **(A)** Expression of E3-ligases in *mib1*$^{-}$ background via *ci-GAL4* in the anterior compartment of wing imaginal discs. Dneur and Neurl1 and NEURL1B are similarly expressed and localize at the plasma membrane. **(B)** Staining of HA-tagged Hybrid ligands in wt background shows similar expression levels and localization at the membrane. **(C)** Quantification of ectopic Wg expression upon ligand over-expression. Hybrid ligands expressed in the ptc domain in *wt* discs. While Dl-DLL1, Dl-DLL4 and Dl-JAG1 show comparable activation to Dl, activation by Dl-JAG2 is significantly weaker. p-values measured by Mann-Whitney U test (n=5). **(D)** Hybrid ligands expressed via *ptc-GAL4* in *mib1*$^{-}$ background fail to induce over-proliferation of the wing pouch, nor activate the endogenous target gene Wg.

Appendix Figure S2

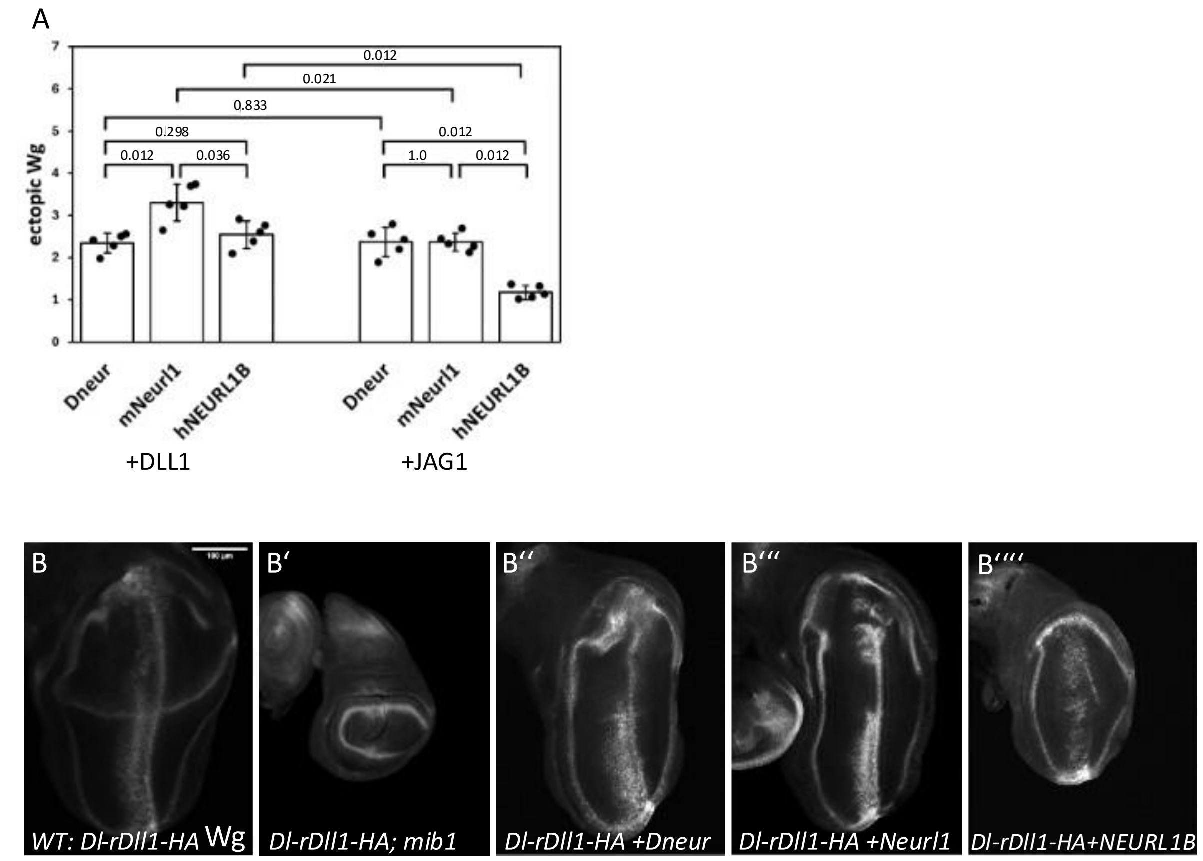

Appendix Fig. S2 (A) Quantification of ectopic Wg activation from Fig. 3. Neurl1/NEURL1B co-expressed with DLL1 induces more ectopic Wg expression than co-expressed with JAG1. NEURL1B co-expression leads to less Wg expression than Neurl1. p-values measured by Mann-Whitney U test (* - $p<0.05$, $n=5$). **(B)** Rat Dll1-ICD shows comparable activation of Wg compared to human DLL1-ICD, underscoring the robustness and reproducibility of the in vivo trans-activation assay.

Appendix Figure S3

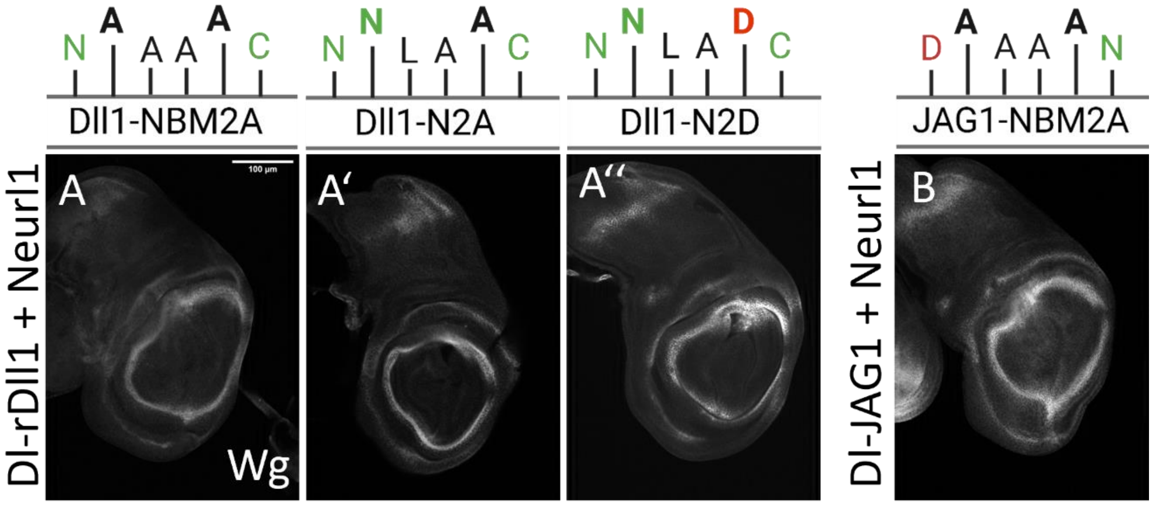

Appendix Fig. S3 (A-A'') Mutation of the NBM in rDll1 leads to complete loss of ectopic Wg expression when co-expressed with Neurl1. Mutation of the second N of the NxxN motif by either A or D leads to a comparable outcome, indicating that the mutation of a single Asparagine within the NBM abolishes activation by Neurl1. **(B)** Complete mutation of the first NxxN motif in JAG1 resembles the point mutation (Fig. 3C'').

Appendix Figure S4

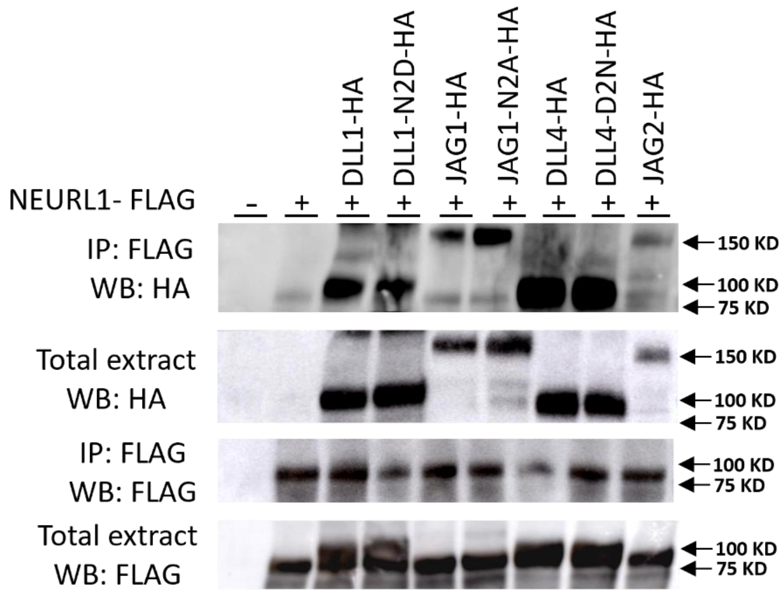

Appendix Fig. S4 The NBM is not required for co-immunoprecipitation of NEURL1 and Notch ligands. NEURL1-mCherry-FLAG (NEURL1-FLAG, 87kD) and HA tagged WT and mutant ligands (DLL1-mTQ2-HA,106kD, DLL4-mTQ2-HA, 102kD, JAG1-mTQ2-HA, 160kD, JAG2-mTQ2-HA, 163kD) were co- expressed in U2Os cells. IP was performed on the indicated cell lysates using anti-FLAG antibodies. Co-IP of HA-ligands was detected by probing the blots with anti-HA antibodies. FLAG antibodies were used to detect the immunoprecipitated NEURL1-FLAG. Total extracts were immunoblotted with anti-HA or ant-FLAG antibodies. The results have been repeated n=3 times.

Appendix Figure S5

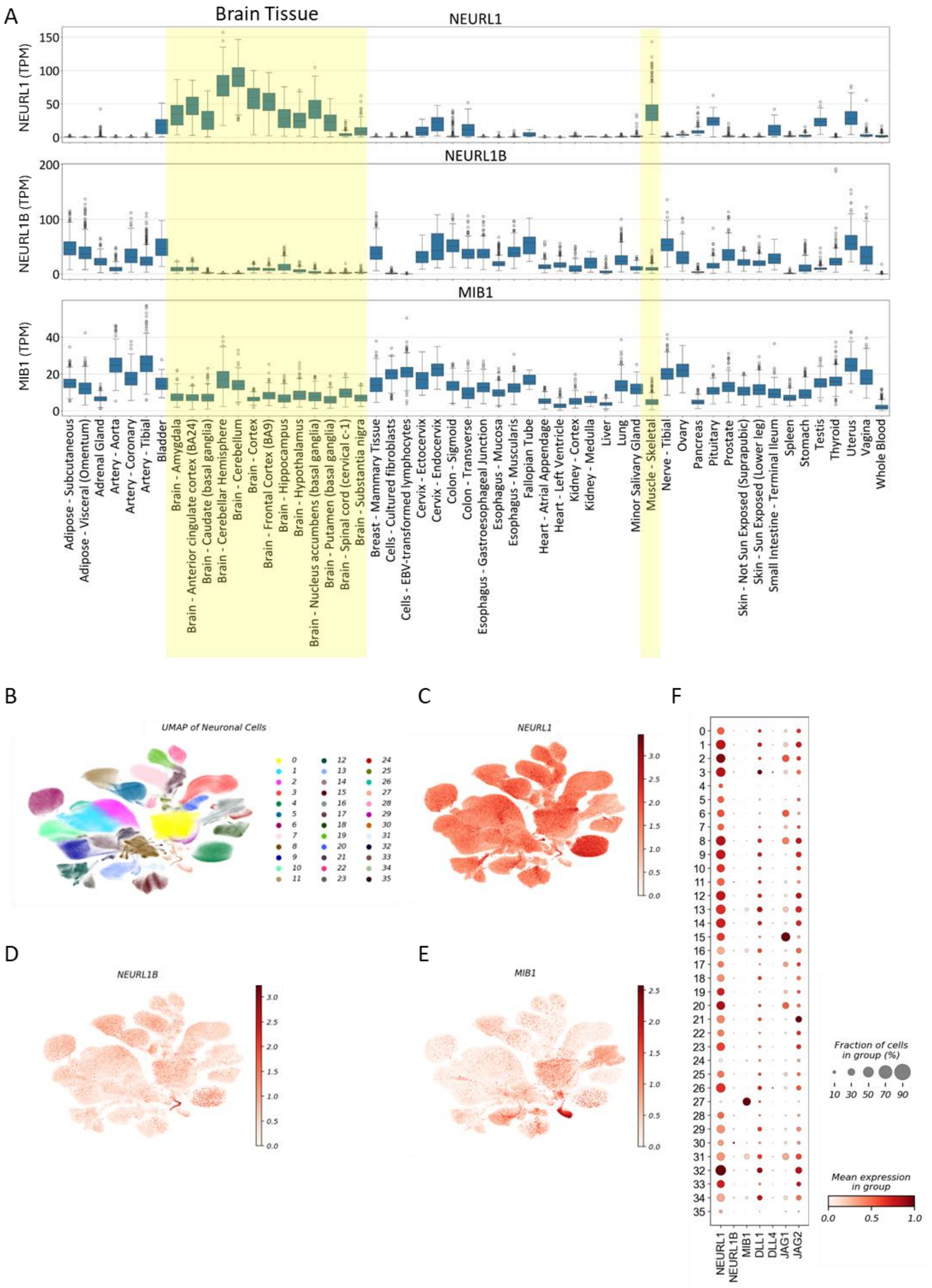

Appendix Fig. S5. NEURL proteins and Mib1 are differentially expressed in different human tissues. Boxplots summarizing the tissue-specific RNA expression levels (nTPM) of NEURL1, NEURL1B, and MIB1 from the GTEx dataset across 54 human tissues. Highlighted are brain tissues and skeletal muscle tissue where NEURL1 expression is notably higher compared to MIB1 and NEURL1B, indicating tissue-specific roles. (B-E) Publicly available single-cell RNAseq data from the Allen Brain Atlas was analyzed and visualized using Uniform Manifold Approximation and Projection (UMAP). (B) UMAP plot identifying 36 neuronal cell clusters based on gene expression profiles. (C-E) Expression intensity of NEURL1 (C), NEURL1B (D), and MIB1 (E) across neuronal clusters, visualized as feature plots. Color intensity represents relative gene expression levels (log-normalized). (F) shows a Dot plot summarizing quantitative analysis of expression for NEURL1, NEURL1B, MIB1, and Notch pathway ligands DLL1, DLL4, JAG1, and JAG2 across neuronal cell clusters. Dot size indicates the fraction of cells expressing each gene within the cluster, while color intensity represents mean expression level.
