# Supplementary material for: Neuralized-like proteins differentially activate Notch ligands
Source: EMBO Rep. 2025 Oct 31;26(23):5756–75. doi: 10.1038/s44319-025-00601-7 (PMC12678834; doi:10.1038/s44319-025-00601-7)
Supplement: Supplementary file 13 — Expanded View Figures [file 44319_2025_601_MOESM13_ESM.pdf]

# Expanded View Figures

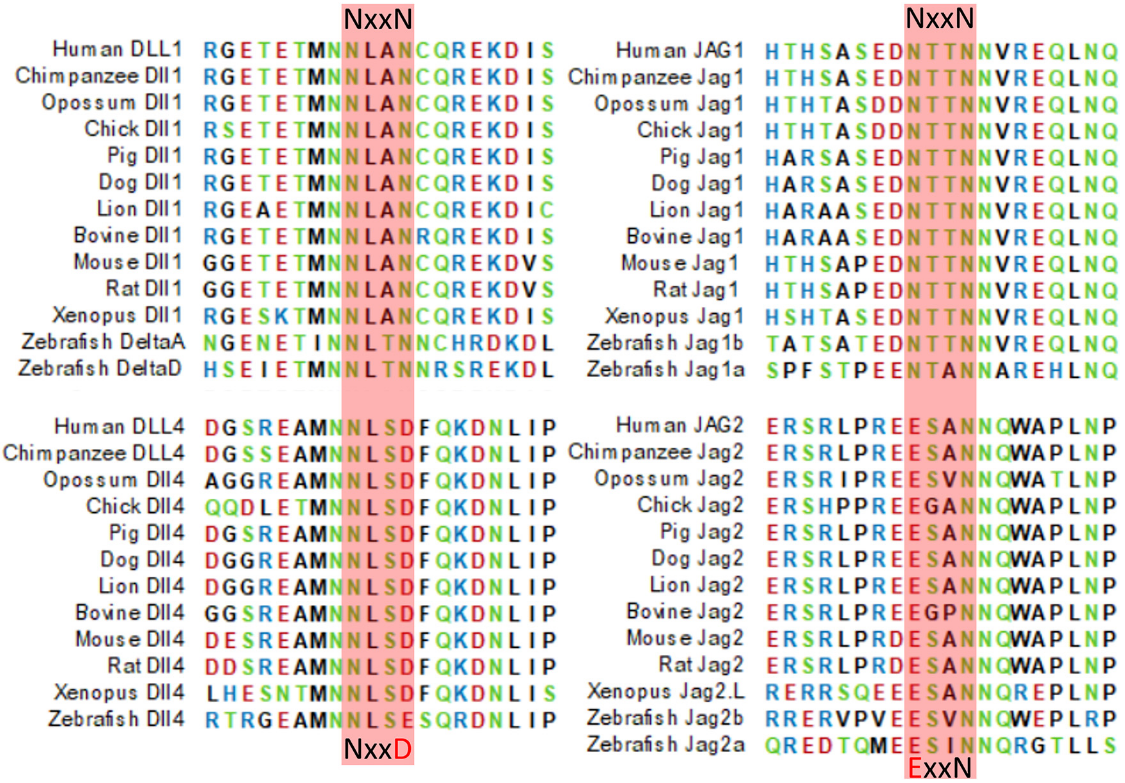


**Figure EV1. Partial sequence comparison of Notch ligands.**

A potential NBM is present in model organisms such as *Xenopus*, *Zebrafish* and *Mouse*, as well as several other species. In DLL1 and JAG1, a conserved NxxN motif is present, while in DLL4 and JAG2, a cryptic motif is present, which contains a D/E instead of an N. These motifs are all located at the N-terminus of the ICDs close to the plasma membrane
